# Effects of Transport Duration and Environmental Conditions in Winter or Summer on the Concentrations of Insulin-Like Growth Factors and Insulin-Like Growth Factor-Binding Proteins in the Plasma of Market-Weight Pigs

**DOI:** 10.3389/fendo.2018.00036

**Published:** 2018-02-13

**Authors:** Elisa Wirthgen, Sébastien Goumon, Martin Kunze, Christina Walz, Marion Spitschak, Armin Tuchscherer, Jennifer Brown, Christine Höflich, Luigi Faucitano, Andreas Hoeflich

**Affiliations:** ^1^Institute of Genome Biology, Leibniz-Institute for Farm Animal Biology (FBN), Dummerstorf, Germany; ^2^Department of Ethology, Institute of Animal Science, Prague, Czechia; ^3^Institute of Genetics and Biometry, Dummerstorf, Leibniz-Institute for Farm Animal Biology (FBN), Dummerstorf, Germany; ^4^Prairie Swine Centre, Saskatoon, SK, Canada; ^5^Ligandis UG, Gülzow-Prüzen, Germany; ^6^Sherbrooke Research and Development Centre, Sherbrooke, QC, Canada

**Keywords:** animal welfare, stress hormones, insulin-like growth factor, pig shipment, metabolism, biomarker

## Abstract

In previous work using market-weight pigs, we had demonstrated that insulin-like growth factors (IGFs) and insulin-like growth factor binding proteins (IGFBPs) are regulated during shipment characterized by changing conditions of stress due to loading or unloading, transportation, lairage, and slaughter. In addition, we found in a previous study that IGFBP-2 concentrations were lower in pigs transported for longer periods of time. Therefore, we performed a more detailed study on the effects of transport duration and season on the plasma concentrations of IGFs and IGFBPs in adult pigs. For the study, exsanguination blood was collected from 240 market-weight barrows that were transported for 6, 12, or 18 h in January or July. IGF-I and -II were detected using commercial ELISAs whereas IGFBPs were quantified by quantitative Western ligand blotting. In addition, established markers of stress and metabolism were studied in the animals. The results show that plasma concentrations of IGFBP-3 were significantly reduced after 18 h of transport compared to shorter transport durations (6 and 12 h; *p* < 0.05). The concentrations of IGF-I in plasma were higher (*p* < 0.001) in pigs transported 12 h compared to shorter or longer durations. Season influenced plasma concentrations of IGFBP-3 and IGF-II (*p* < 0.05 and *p* < 0.01, respectively). Neither transport duration nor differential environmental conditions of winter or summer had an effect on glucocorticoids, albumin, triglycerides, or glucose concentrations (*p* > 0.05). However, low-density lipoprotein concentrations decreased after 18 h compared to 6 h of transport (*p* < 0.05), whereas high-density lipoprotein concentrations were higher (*p* < 0.05) in pigs transported for 12 or 18 h compared to those transported for only 6 h. Our findings indicate differential regulation of IGF-compounds in response to longer transport duration or seasonal changes and support current evidence of IGFs and IGFBPs as innovative animal-based indicators of psycho-social or metabolic stress in pigs.

## Introduction

Due to concentration of the slaughter process, more pigs are being transported to less and bigger slaughter plants resulting in increasingly longer transport distances and lengths (1). Longer transport distance or duration is associated with increased loss of live-weight (2), mortality (3), serum concentrations of acute phase proteins (4), creatine kinase (CK) (5, 6), glucose, lactate, and hematocrit levels (7, 8). The collection of exsanguination blood directly after the slaughtering of pigs is a suitable, non-invasive technique for the evaluation of the physiological response to different transport durations and preslaughter conditions. As described in preliminary studies (9), cortisol concentrations, measured in exsanguination blood, were not affected after prolonged duration of shipment. Though, insulin-like growth factor-binding protein (IGFBP)-2 concentrations decreased over time due to the length of shipment indicating biomarker potential for components of insulin-like growth factor (IGF) system. Furthermore, IGFBP-3/-2 ratio was increased in pigs, which were repeatedly stressed in the period of 24 h before slaughter. In humans, parameters of the growth hormone (GH) axis may have biomarker potential for acute or prolonged illness (10, 11). In mammals, it is assumed that acute physical stress, energy restriction, or acute phase of severe illness induce an amplification of GH secretion and increased levels of GH (10, 12, 13). GH affects body growth and metabolism directly and indirectly *via* control of IGF-I production in the liver or in other tissues (14). In blood, IGF is bound to IGFBPs that control the availability of IGF, but also have IGF-independent functions (15). As IGFBPs are sensitive markers to detect changes of the GH-dependent growth (16, 17), they play a central role in linking nutritional intake with somatic growth (18–20). Furthermore, it is known that glucocorticoids influence the levels of IGF-I and IGFBPs (21–23) suggesting an interference of acute or prolonged stress with the IGF system. Accordingly, in the present study, effects of transport duration under different seasonal conditions on the IGF-system were discussed in conjunction with established stress markers.

## Materials and Methods

All experimental procedures were approved by the University of Saskatchewan’s Animal Research Ethics Board and adhered to the current guidelines of the Canadian Council on Animal Care (CCAC, 2009).

### Animals and Preslaughter Conditions

Details about the experimental conditions applied in this experiment were previously described by Goumon et al. (24). Briefly, this experiment was part of a larger study involving 5,040 crossbred pigs (*Sus scrofa*, Landrace × Large White; mean body weight = 120.8 ± 0.4 kg) transported over 6, 12, or 18 h (average loading density of 0.37–0.38 m^2^/pig) to a slaughterhouse located in western Canada. This study was conducted with subset of 240 male pigs (barrows, 120.8 ± 0.4 kg) at the age about 24 weeks and includes two trials. One trial was conducted in January to February with a temperature range between −28.8 and 1.9°C during the transport. The second trial was conducted in July and the temperature ranged between 12.5 and 40.1°C. Food was removed from pigs transported for 6 and 12 or 18 h for 20 and 24 h, respectively. As previously described, fasting did not affect the circulating levels of IGF-1 and IGFBPs within the time frame of this experiment (9). The pigs had no access to water on the truck, but water was available in lairage. After unloading, pigs were held in a lairage for approximately 150 min.

### Blood Collection

At exsanguination, 2 ml of blood were collected from a subsample of 240 barrows (40 barrows/transport duration/season) and EDTA-plasma was extracted (centrifugation at 1,400 × *g* at 4°C for 12 min) (24). Plasma samples were stored at −80°C before shipment to the laboratory of the Institute of Genome Biology at the Leibniz Institute for Farm Animal Biology (FBN) in Dummerstorf (Germany) for further analyses.

### Plasma Analyses

#### Insulin-Like Growth Factor Binding Proteins

Quantitative Western ligand blotting was applied for the assessment of IGFBP-3, -2 and -5 concentrations as described previously (25). Briefly, plasma samples were denatured for 5 min in sample buffer (312.5 mM Tris (pH 6.8), 50% (w/v) glycerol, 5 mM EDTA, 1% (w/v) SDS, and 0.02% bromophenol blue). After separation by 12% SDS-PAGE, proteins were transferred to a polyvinylidene fluoride membrane (Millipore, Bedford, MA, USA), blocked and incubated with biotin labeled human IGF-II (1:500; BioIGF2-10; ibt-systems, Binzwangen, Germany). IGFBPs were detected by enhanced chemiluminescence using LuminataTM Forte (Millipore, Bedford, MA, USA). The signal intensities were corrected for background using the Gelanalyzer2010a software. On each blot, serial dilutions of recombinant human IGFBP-2 to -5 standards (R & D Systems, Wiesbaden, Germany) in artificial serum matrix (Biopanda, County Down, UK) were used as calibrators enabling signal quantification. Each signal was corrected for unspecific background Curve fitting was achieved by non-linear regression of each separate IGFBP. Due to low abundance, IGFBP-4 was detected but not quantified in the porcine plasma. The analytical range for each plasma IGFBP was 150–15,000 ng/ml. Inter-assay coefficients of variation (CV) were determined by *in study* validation according to recommendations of EMA guideline (26) using a random selected pig plasma sample of the investigated study samples. The inter-assay CVs (*n* = 10) for IGFBP-3 (mean: 4850 ng/ml), -2 (mean: 2324 ng/ml), and -5 (679 ng/ml) were 12.8, 15.1, and 20.1%, respectively.

#### IGF-I and IGF-II

Plasma concentrations of IGF-I and IGF-II (*n* = 90) were analyzed by Ligandis GbR using commercially available ELISA Kits E20 and E30 according to manufacturer’s instruction (Mediagnost, Reutlingen, Germany). For IGF-I, the analytical range was 21–1,050 ng/ml and the inter- and intra-assay CVs were less than 6.8 and 6.7%, respectively. For IGF-II, the analytical range was from 120 to 2,400 ng/ml.

#### Corticosterone

Plasma concentrations of corticosterone (*n* = 90) were analyzed using LC-MS technique as already described (27). In brief, after protein precipitation, the supernatant was dried and stored at −20°C. For LC–MS/MS analysis, samples were dissolved in MeOH/H_2_O (50/50) adding DXM (100 ng/ml), vortexed for 30 s, sonicated for 2 min, and centrifuged at 4°C for 2 min at 14,000 rpm. Subsequently, samples were transferred to mass spectrometry analysis using an Accela HPLC/autosampler system (Thermo Fisher Scientific) coupled to the LTQ Orbitrap high-resolution hybrid mass spectrometer (Thermo Fisher Scientific, Dreieich, Germany). At various concentrations between 5 and 500 ng/ml, the intra-assay CVs were 13.05–4.64%. The inter-assay CV for 100 ng/ml (*n* = 20) was 5.57%.

#### Metabolites

Triglycerides (TG), cholesterol, glucose, high-density lipoprotein cholesterol (HDL-C), and albumin (*n* = 90) were analyzed in plasma using commercial enzymatic colored kits according to the manufacturer’s instructions (TG: No. LT-TR 1002, total cholesterol: No. LT-CH 0503, glucose: LT-GL 0251, HDL: LT-HD 0053, albumin LT-AB 0103; Labor & Technik Eberhard Lehmann Berlin, Germany, respectively) as previously described (28). The intra-assay CV for TG, total cholesterol, glucose, HDL and albumin were 1.60, 1.32, 1.80, 2.44, and 1.66%, respectively. The inter-assay CV% for TG, total cholesterol, glucose, HDL, and albumin were 4.30, 1.98, 2.40, 2.50, and 1.11%, respectively. Low-density lipoprotein cholesterol (LDL-C) was calculated according to the Friedewald Formula = [total cholesterol] − [HDL-C] − ([TG]/5) (29, 30).

### Statistics

Statistical analyses were performed using SAS software version 9.3 (SAS, Cary, NC, USA). The data of all blood parameters were evaluated by ANOVA using the MIXED procedure. The ANOVA model included transport duration (6, 12, and 18 h), environment (January/February versus July), week within environment (subset of weeks 1, 2, 3, and 4 in January/February, subset of weeks 1, 2, 3, and 4 in July) and the two-way interaction duration × environment as fixed effects. For all data, the least squares means and their SE were calculated and tested for each fixed effect in the model using the Tukey–Kramer procedure for all pair-wise multiple comparisons. Effects and differences were considered significant if *p* < 0.05.

## Results

### Effect of Transport Duration

**Figure 1:**
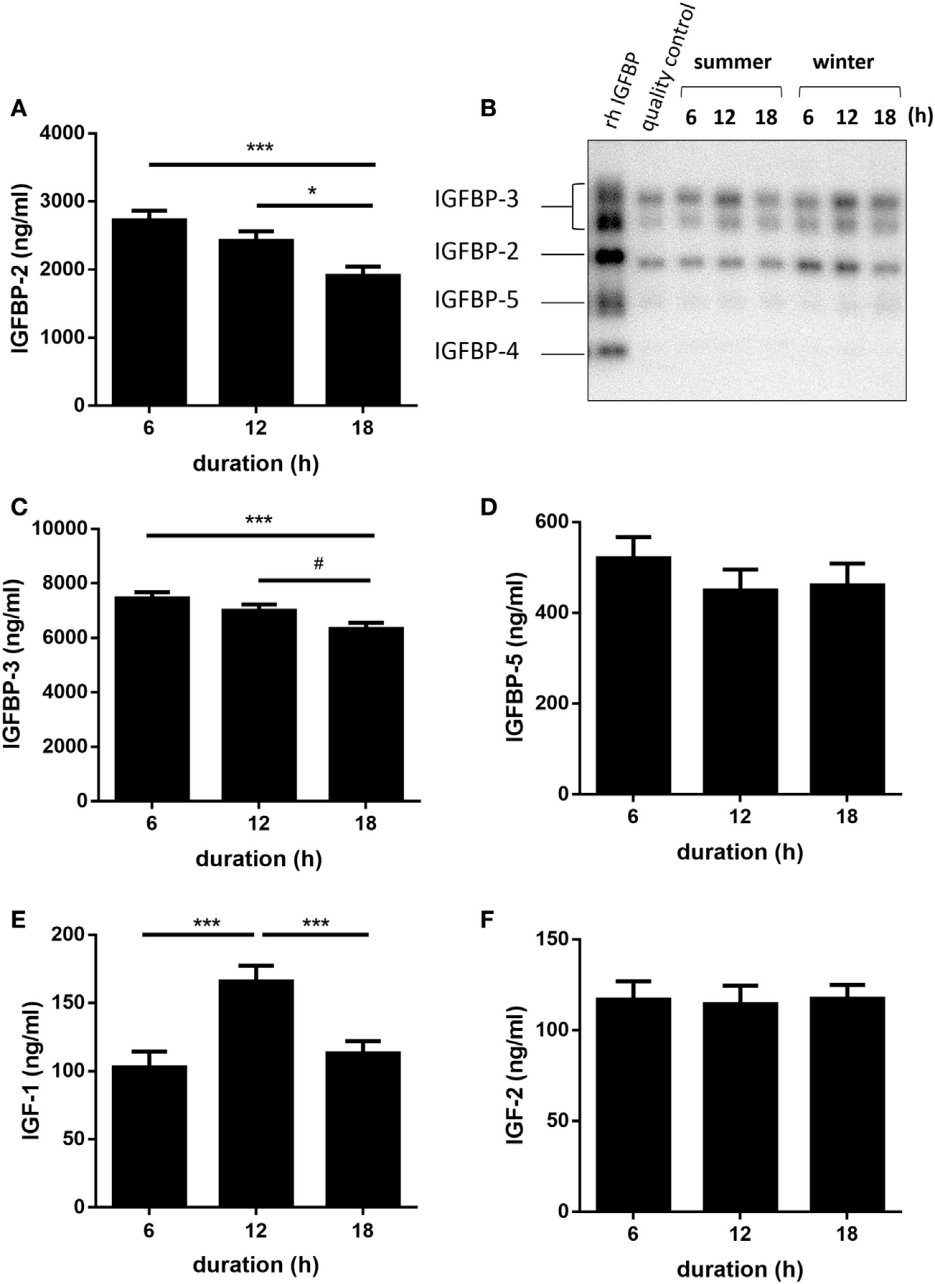
Effect of transport duration on insulin-like growth factor (IGF)-axis: insulin-like growth factor-binding protein (IGFBP)-2 **(A)**, with kind permission from Nature Publishing Group as previously published in Ref. (9), IGFBP-profile **(B)**, IGFBP-3 **(C)**, IGFBP-5 **(D)**, IGF-I **(E)**, and IGF-II **(F)**. Quantitative data are presented as LS-Means + SE and include the summarized data of the trials in January/February and July due to the absence of the significant interaction duration × environment (*p < 0.05, **p < 0.01, ***p < 0.001, #*p* < 0.1, rh: recombinant human, quality control: pig plasma sample). IGFBP-2, -3, -5: *n* = 80 per duration; IGF-I and IGF-II: *n* = 30 per duration.

**Figure 2:**
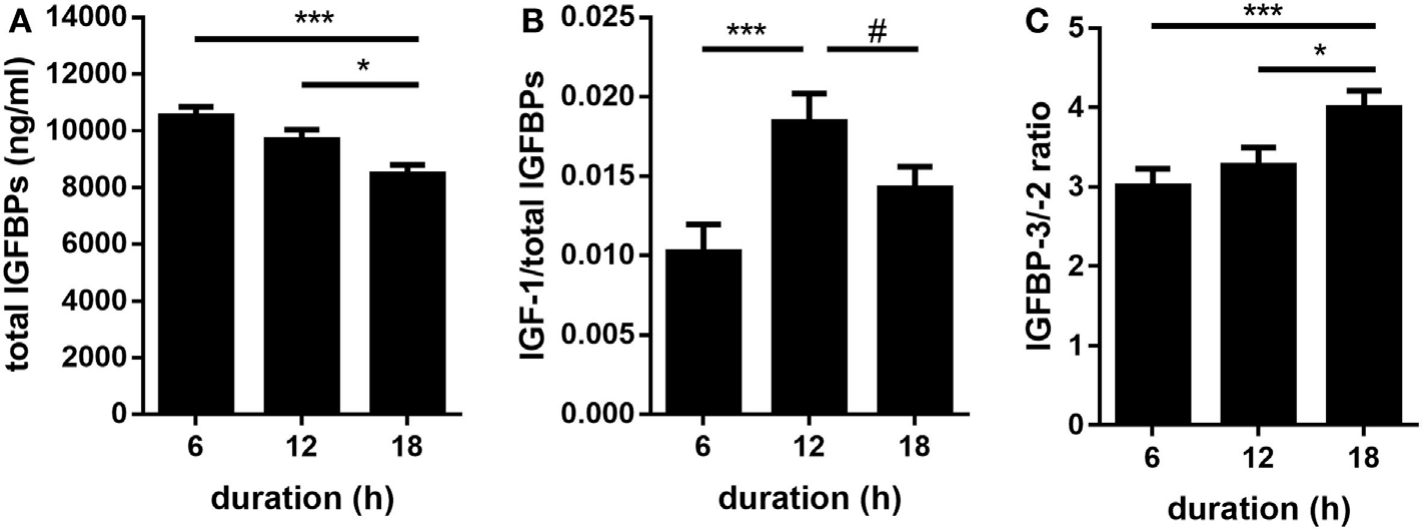
Effect of transport duration on total amount of total insulin-like growth factor-binding proteins [IGFBPs; **(A)**] as a marker for IGF-binding capacity and on the ratio of IGF-I/total IGFBPs **(B)** as indicator for IGF-I bioavailability. IGFBP-3/-2 ratio **(C)** was detected as a marker of growth hormone action. Data are presented as LS-Means + SE. Total **(A,B)**: *n* = 30 per duration, **(C)** *n* = 80 per duration. *p < 0.05, ***p < 0.001, #*p* < 0.1.

An overview of all calculated *F*-values, degrees of freedom, and probability *F*-values of each fixed effect and their interaction is provided by Table 1. Quantitative Western ligand blot analyses of IGFBPs revealed IGFBP-3 as being the most abundant plasma IGFBP in pigs followed by IGFBP-2 and IGFBP-5 (Figures 1A–D) and having similar molecular weights as human recombinant reference standards. Plasma concentrations of IGFBP-3 and IGFBP-2 were lower in pigs transported for 18 h compared to pigs transported for 6 h (*p* < 0.001 and *p* < 0.001, respectively; Figures 1A,C). However, the concentrations of both IGFBP did not differ in pigs transported for 6 and 12 h (*p* > 0.05). There was no significant effect of transport duration on plasma concentrations of IGFBP-5 either (*p* > 0.05; Figure 1D). Concentrations of IGF-I were higher (*p* < 0.001) in pigs transported for 12 h than in those transported for 6 or 18 h (Figure 1E). By contrast, plasma concentrations of IGF-II were not significantly affected by transport duration (*p* > 0.05; Figure 1F). The total amount of quantified IGFBPs, which is an indicator for IGF-binding capacity, was lower in pigs transported for 18 h compared to pigs transported for 6 and 12 h (*p* < 0.001 and *p* < 0.05, respectively; Figure 2A). The ratio of IGF-I to total IGFBPs, calculated as an indicator for IGF-I bioavailability in circulation, was greater (*p* < 0.001) in pigs transported for 12 h compared to those transported for 6 h (Figure 2B). As shown in Figure 2C, the ratio of IGFBP-3 to -2, used as a marker for somatic growth and metabolic homeostasis, was greater in pigs transported for 18 h compared to pigs transported for 6 or 12 h (*p* < 0.001 and *p* < 0.05, respectively).

**Table 1:** *F*-test for the fixed effects season, week within season, duration, and the interaction of environment × duration.

Parameter	Effect	NumDF	DenDF	*F*-value	ProbF
IGFBP-3	Environment	1	228	5.50	**0.020**
	Week (environment)	6	228	1.61	0.147
	Duration	2	228	8.01	**<0.001**
	Environment × duration	2	228	0.26	0.768

IGFBP-2	Environment	1	225	0.02	0.892
	Week (environment)	6	225	1.62	0.143
	Duration	2	225	11.30	**0.001**
	Environment × duration	2	225	2.05	0.131

IGFBP-5	Environment	1	154	0.46	0.500
	Week (environment)	6	154	1.18	0.322
	Duration	2	154	0.79	0.455
	Environment × duration	2	154	0.48	0.622

IGF-I	Environment	1	81	0.55	0.460
	Week (environment)	3	81	2.84	**0.043**
	Duration	2	81	15.51	**<0.001**
	Environment × duration	2	81	2.22	0.115

IGF-II	Environment	1	81	7.82	**0.006**
	Week (environment)	3	81	1.31	0.277
	Duration	2	81	0.04	0.962
	Environment × duration	2	81	1.86	0.163

total IGFBPs	Environment	1	228	3.22	0.074
	Week (environment)	6	228	1.59	0.151
	Duration	2	228	11.50	**<0.001**
	Environment × duration	2	228	0.41	0.664

IGF-I/total GFBPs	Environment	1	81	0.30	0.585
	Week (environment)	3	81	1.29	0.284
	Duration	2	81	10.22	**<0.001**
	Environment × duration	2	81	2.13	0.126

IGFBP-3/IGFBP-2	Environment	1	225	1.67	0.198
	Week (environment)	6	225	0.50	0.809
	Duration	2	225	6.66	**0.001**
	Environment × duration	2	225	1.87	0.157

Corticosterone	Environment	1	81	0.19	0.663
	Week (environment)	3	81	1.17	0.325
	Duration	2	81	0.09	0.915
	Environment × duration	2	81	1.78	0.176

Glucose	Environment	1	74	0.94	0.334
	Week (environment)	3	74	2.14	0.103
	Duration	2	74	0.26	0.772
	Environment × duration	2	74	0.13	0.880

Albumin	Environment	1	75	2.64	0.108
	Week (environment)	3	75	1.67	0.180
	Duration	2	75	2.13	0.126
	Environment × duration	2	75	0.79	0.456

Triglycerides	Environment	1	81	0.00	0.951
	Week (environment)	3	81	1.55	0.208
	Duration	2	81	8.38	**<0.001**
	Environment × duration	2	81	4.88	**0.010**

Cholesterol	Environment	1	81	3.35	0.071
	Week (environment)	3	81	0.45	0.719
	Duration	2	81	5.16	**0.008**
	Environment × duration	2	81	1.87	0.160

HDL-C	Environment	1	81	1.05	0.308
	Week (environment)	3	81	0.17	0.915
	Duration	2	81	11.67	**<0.001**
	Environment × duration	2	81	1.71	0.188

LDL-C	Environment	1	81	2.67	0.106
	Week (environment)	3	81	0.64	0.588
	Duration	2	81	6.06	**0.003**
	Environment × duration	2	81	0.89	0.415

*(NumDF, numerator degrees of freedom; DenDF, denominator degrees of freedom; ProbF, significance probability corresponding to the *F*-value)*.

*IGFBP, insulin-like growth factor-binding protein; IGF, insulin-like growth factor; HDL, high-density lipoprotein; LDL, low-density lipoprotein*.

*ProbF < 0.05 were considered significant and marked with bold font*.

**Figure 3:**
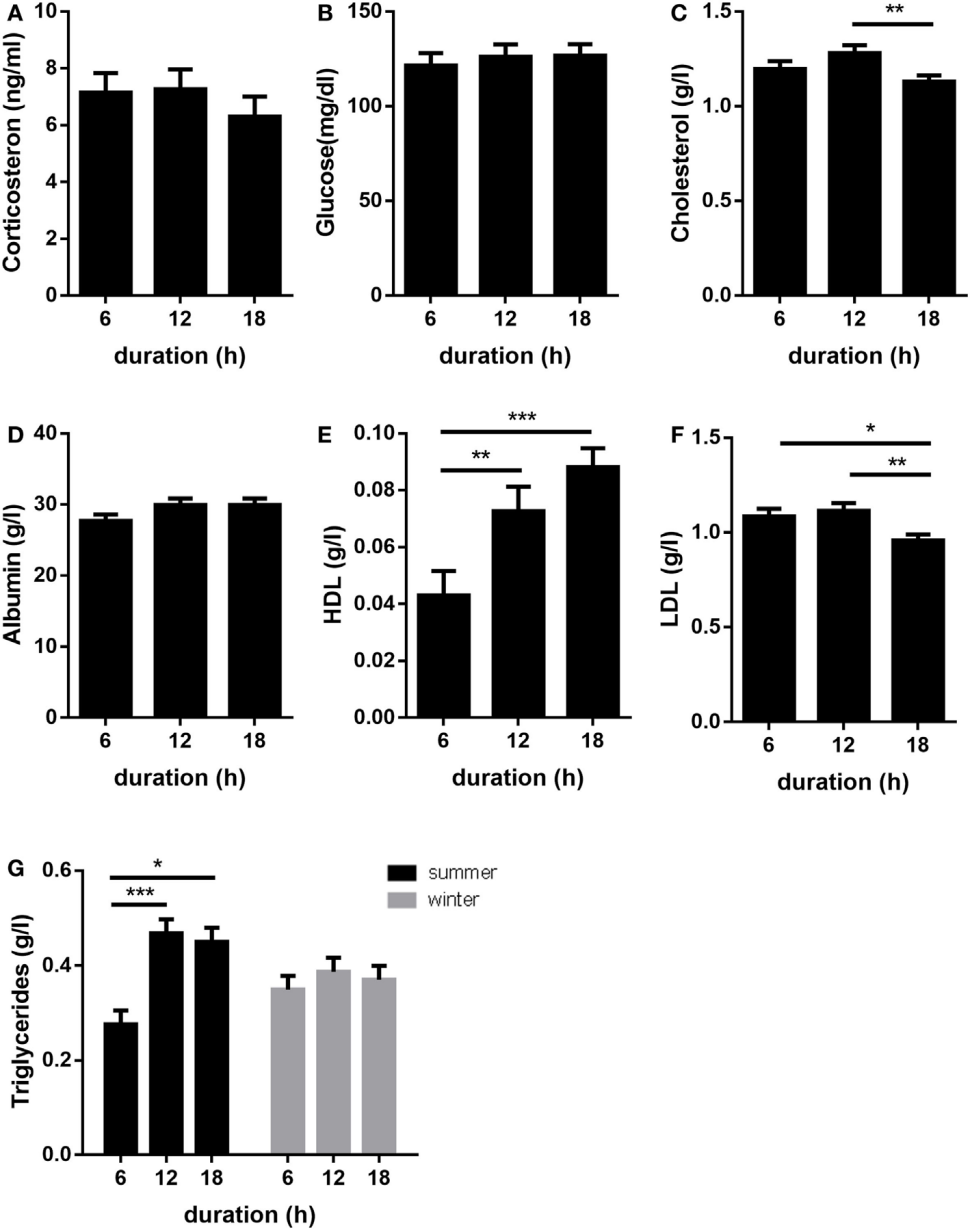
Effects of transport duration on plasma levels of corticosterone **(A)**, glucose **(B)**, albumin **(C)**, cholesterol **(D)**, HDL **(E)**, LDL **(F)**, and triglycerides **(G)** (*n* = 28–30 per duration for all parameters). Data except triglycerides include the summarized data of the trials in January/February and July due to the absence of the significant interaction duration × environment. A significant interaction of duration × environment (*p* < 0.05) was calculated for TG. All data are presented as LS-Means + SE *p < 0.05, **p < 0.01, ***p < 0.001; LDL = low-density lipoprotein, HDL = high-density lipoprotein.

Plasma concentrations of corticosterone, glucose, and albumin were not significantly affected by transport duration (*p* > 0.05; Figures 3A–C). Plasma concentrations of total cholesterol were lower (*p* < 0.01) in pigs transported for 18 h compared to those transported for 12 h (Figure 3D). When compared to 6 h transports, HDL-cholesterol levels were greater after 12 h (*p* < 0.01) and 18 h (*p* < 0.001) transports (Figure 3E). By contrast, LDL-cholesterol concentrations were lower in pigs transported for 18 h compared to pigs transported for 6 or 12 h (*p* < 0.05 and *p* < 0.01, respectively; Figure 3F). An effect of transport duration on plasma TG was only detected in summer. Thereby, the concentrations of TG were increased after 12 and 18 h transport compared to 6 h (*p* < 0.001 and *p* < 0.05, respectively, Figure 3G).

### Effect of Differential Environment in Winter or Summer

The environmental conditions had an effect on some plasma parameters in this study, with concentrations of IGFBP-3 being higher (*p* < 0.05) in January/February than in July and IGF-II concentrations being lower (*p* < 0.01) in January/February than in July (Table 2). Plasma IGFBP-2, IGFBP-5, IGF-I, cortisol, corticosterone, albumin, and glucose concentrations were not affected by the differential environmental conditions in this study (*p* > 0.05).

**Table 2:** Effect of different environmental conditions in January/February and July on plasma parameters.

	January/February	July	*n*_total_	*p*-Value
IGFBP-3 (ng/ml)	7,295 ± 246	6,559 ± 194	240	**0.02**
IGFBP-2 (ng/ml)	2,364 ± 150	2,338 ± 122	240	0.89
IGFBP-5 (ng/ml)	461 ± 53.0	414 ± 44.0	240	0.50
IGF-1 (ng/ml)	133 ± 13	121 ± 8.91	90	0.46
IGF-2 (ng/ml)	97.1 ± 11.3	135 ± 7.72	90	**0.01**
Corticosterone (ng/ml)	6.3 ± 1.07	6.8 ± 0.73	90	0.66
Albumin (g/dl)	3.1 ± 0.10	2.9 ± 0.11	84	0.11
Glucose (mg/dl)	129 ± 6.02	120 ± 6.71	84	0.33
Total IGFBPs	9,981 ± 374	9,125 ± 295	240	0.07
IGF-1/total IGFBP	0.01 ± 0.002	0.01 ± 0.001	90	0.58
IGFBP-3/IGFBP-2	3.6 ± 0.24	3.22 ± 0.20	240	0.20
Total cholesterol (g/l)	1.15 ± 0.04	1.25 ± 0.03	90	0.07
HDL (g/l)	0.1 ± 0.01	0.07 ± 0.01	90	0.31
LDL (g/l)	1.0 ± 0.04	1.09 ± 0.03	90	0.11

*For all presented parameters the interaction duration × environment was not significant. The data are summarized over the transport duration (6, 12, and 18 h) and are presented as LS-means ± SE. The Tukey–Kramer procedure was used for pair-wise comparisons*.

*ProbF < 0.05 were considered significant and marked with bold font*.

*IGFBP, insulin-like growth factor-binding protein; IGF, insulin-like growth factor; HDL, high-density lipoprotein; LDL, low-density lipoprotein*.

## Discussion

The results of this study clearly demonstrate that transport duration and differential environmental conditions in winter or summer contribute to the variation of IGFs and IGFBPs in blood collected from pigs at slaughter. Indeed, the concentrations of IGFBP-3 were lower after 18 h transport, contributing also to reduction of total IGFBPs levels in the circulation. Due to a stronger reduction of IGFBP-2, the ratio of IGFBP-3/IGFBP-2 increased after 18 h of transportation. An increase of IGFBP-3/IGFBP-2 ratio was also reported in plasma of pigs subjected to repeated restraint stress in the preslaughter period (9) indicating that this ratio might reflect increased stress conditions before slaughtering. In fact, in a previous study (24), 18 h of transportation were considered as a more stressful condition because the animals were characterized by higher body temperatures or greater drinking and differential resting behavior, compared to 6 or 12 h transports. In the present study, pigs were fasted either 20 h (6 and 12 h transport) or 24 h (18 h transport). Because it has been shown, that fasting of 70 h reduced serum levels of IGF-I and IGFBP-3 (31) in sheep, it might be that the IGF-compounds, at least in theory, were coregulated by metabolic stress in pigs. However, preliminary studies revealed that 19 h of fasting were not sufficient to affect IGFBP-2, -3, or -5 concentrations also in market-weight pigs (9). Furthermore, circadian effects as described for IGFBP-3 (9) are no explanation for the decrease of IGFBP-3 due to the same time of arrival of animals at the slaughterhouse. Therefore, together with the findings from preliminary studies, the reduction of plasma IGFBP-2 and IGFBP-3 levels in two independent conditions of physiological and psycho-social stress in pigs, such as restraint and long transport, provides consistent evidence for the potential of both IGFBPs as stress biomarker in pigs.

Interestingly, in the present study, IGF-I concentrations and IGF-I bioavailability were increased after 12-h transport followed by a decrease after 18 h to levels similar to those after 6 h. Since the amount of IGFBPs present in the circulation was not significantly different after 6 and 12 h of transport, it is difficult to conclude whether the IGFBPs are responsible for the increased IGF-I plasma concentrations after 12 h transport. Nevertheless, altered biosynthesis of IGF-I might be assessed by follow-up studies in hepatic and non-hepatic tissues. Without excluding a possibly reduced expression of IGF-I during 18 h transport, the reduced levels of IGFBPs may explain the reduction of IGF-I concentrations in pig plasma after long-term transport due to reduced half-life of free IGF-I.

In the present study, IGFBP-3 concentrations were higher in pigs transported in January/February, while IGF-II concentrations were higher in pigs transported in July. This might be an effect of environmental conditions such as different temperatures during the transport or husbandry. To our knowledge, there is little evidence regarding the effect of ambient conditions on the IGF-system in farm animals and, before this study, there was none for pigs. In fish, it has been shown that plasma and mRNA levels of IGF-I increase with ambient water temperature, while IGF-II concentrations decrease (32). In the Gabillard et al. (2003) study, ambient temperature variation appears to promote fish growth through IGF-I secretion by the liver following GH stimulation. However, this effect was biased by the fish nutritional condition. By contrast, plasma IGF-II was not affected by the growth-promoting effect of temperature, but appeared to be more related to the metabolic status of the fish. A positive correlation of circulating levels of IGF-1 with increased water temperatures was also described in other fish species (33–36). Differently from the findings in fish, no significant changes in IGF-I levels were found in this study. A possible explanation for this lack of effect may be that IGF-I action was modulated by the increased levels of IGFBP-3, which was by the way the most abundant IGFBP in pig serum in this study, at the conditions of lower temperatures. In addition, regarding the thermoregulation, most fish species are poikilothermic ectotherms. This means, the body temperature is not constant but varies with water temperature which may have direct effects on metabolic and endocrine parameters in contrast to endothermic mammals. Contrary to the results of the fish study (32), IGF-II level variation was not inversely proportional to that in ambient temperature in the present study, probably due to the potential negative effect of heat stress on the expression of growth-related genes (37). It has been also suggested that potential environmental effects on IGF-system in market pigs are strongly dependent on the farm management system. This hypothesis also needs to be assessed in future studies.

Longer transport duration and/or extreme ambient temperatures are known to decrease the welfare of pigs on the truck (3, 24, 38–40). However, the transport times either applied during differential conditions in winter or summer in this study did not result in any variation in the concentrations of corticosterone, glucose, and albumin in exsanguination blood. This is in accordance to previous findings that cortisol was affected by the transport duration (6, 9). The lack of variation in these blood metabolites at slaughter may indicate that lairage conditions and time applied in this study were sufficient to help pigs recover from transport stress, regardless of travel time. A number of studies (41–43) actually reported that during two or 3 h in lairage, levels of blood cortisol are normalizing.

Higher plasma total protein and albumin concentrations in blood of pigs at exsanguination are associated with the dehydration rate at the time of slaughter (44, 45). It is sensible that pigs are getting dehydrated during extended periods of shipment, which is in line with greater drinking behavior in pigs transported longer versus shorter periods of time (41, 46). However, in the present study, no effects of transport duration or environment were observed on blood albumin levels at slaughter. The increased drinking behavior observed in lairage in pigs after a transport period of 18 h in a previous study (24) may have helped circulating levels of albumin to return to the rest ones.

Differently from glucocorticoids and albumin, the indicators of lipid metabolism in the present study were affected by transport duration and/or environment. Plasma lipid levels may reflect constitutional or nutritional status of an animal, but there is also some evidence that plasma lipid concentrations can also be affected by short-term emotional arousal (47), acute stress (48), or activation of inflammatory pathways (49). In the present study, total cholesterol was reduced after 18 h of transport and this reduction was due to the simultaneous reduction of LDL-C concentrations. Conversely, HDL-C concentrations increased in pigs transported for 12 and 18 h. Transport longer than 6 h also increased blood TG levels, but only in summer. These results may be explained by the concurrent effects of longer feed deprivation (24 h) and transport (18 h) on increased lipolysis or even protein degradation for maintenance of metabolic homeostasis as showed by the reduced levels of cholesterol and LDL-C in this study, and the variation of body temperature and post-transport resting behavior reported in a companion study (24). However, as in 24 h fasted pigs, live-weight reductions are possibly related to excretion (50, 51) and considering that the preslaughter fasting intervals between transport groups were almost identical (i.e., 20 h fasting in the 6 and 12 h groups, and 24 h of fasting in the 18 h group), preslaughter lipid metabolism may have been affected by the length of transport rather than fasting time in this study. Warriss, in fact, considered the possibility that shipment might have a stronger effect on reductions of live-weight than fasting alone (52).

To summarize and conclude, in this study the IGF-system was regulated under conditions of different transport durations. With prolonged transport, plasma IGFBP-2 and IGFBP-3 levels were reduced, whereas IGF-I concentrations were dynamically regulated with increased concentrations after intermediate transport duration (12 h) compared to shorter or extended periods of time (6 or 18 h). By contrast, based on the results of this study, glucocorticoids, glucose, or albumin cannot be considered as useful indicators of transport stress in pigs. The metabolic stress condition of pigs being transported for 18 h was also indicated by the reduced levels of cholesterol and LDL-C and was associated with a decline of plasma IGFBPs concentrations. Further studies on the differential effects of psycho-social or metabolic stress on IGFs, IGFBPs, and complex traits of IGF-related signatures in pigs might identify specific biomarker potential for compounds from the IGF-system for animal welfare.

## Ethics Statement

All experimental procedures were approved by the University of Saskatchewan’s Animal Research Ethics Board and adhered to the current guidelines of the Canadian Council on Animal Care (CCAC, 2009).

## Author Contributions

All authors designed the experiments and wrote the manuscript. SG, JB, and LF performed the animal experiment. EW, MK, CW, MS, and CH carried out wet lab experiments. EW, MK, CW, MS, AT, CH, and AH analyzed data.

## Conflict of Interest Statement

CH and AH are related to Ligandis UG. The other authors do not have any potential conflicts of interest to declare.
